# Epidemiological profile of SARS-CoV-2 among selected regions in Ghana: A cross-sectional retrospective study

**DOI:** 10.1371/journal.pone.0243711

**Published:** 2020-12-10

**Authors:** Michael Owusu, Augustina Angelina Sylverken, Sampson Twumasi Ankrah, Philip El-Duah, Nana Kwame Ayisi-Boateng, Richmond Yeboah, Richmond Gorman, Jesse Asamoah, Tabea Binger, Godfred Acheampong, Franklin Asiedu Bekoe, Sally-Ann Ohene, Rita Larsen-Reindorf, Anthony Afum-Adjei Awuah, John Amuasi, Ellis Owusu-Dabo, Yaw Adu-Sarkodie, Richard Odame Phillips

**Affiliations:** 1 Department of Medical Diagnostics, Kwame Nkrumah University of Science and Technology, Kumasi, Ghana; 2 Kumasi Centre for Collaborative Research in Tropical Medicine, Kwame Nkrumah University of Science and Technology, Kumasi, Ghana; 3 Department of Theoretical and Applied Biology, Kwame Nkrumah University of Science and Technology, Kumasi, Ghana; 4 Department of Statistics and Actuarial Sciences, Kwame Nkrumah University of Science and Technology, Kumasi, Ghana; 5 Institute of Virology, Charite, Universitätsmedizin Berlin, Berlin, Germany; 6 Department of Medicine, Kwame Nkrumah University of Science and Technology, Kumasi, Ghana; 7 Centre for Health Systems Strengthening, Kumasi, Ghana; 8 Disease Surveillance Unit, Ghana Health Service, Accra, Ghana; 9 World Health Organisation Office, Accra, Ghana; 10 Ashanti Regional Health Directorate, Ghana Health Service, Accra, Ghana; 11 Department of Molecular Medicine, Kwame Nkrumah University of Science and Technology, Kumasi, Ghana; 12 Department of Global and International Health, Kwame Nkrumah University of Science and Technology, Kumasi, Ghana; 13 Department of Clinical Microbiology, Kwame Nkrumah University of Science and Technology, Kumasi, Ghana; National Institute for Infectious Diseases Lazzaro Spallanzani-IRCCS, ITALY

## Abstract

**Background:**

Global cases of COVID-19 continue to rise, causing havoc to several economies. So far, Ghana has recorded 48,643 confirmed cases with 320 associated deaths. Although summaries of data are usually provided by the Ministry of Health, detailed epidemiological profile of cases are limited. This study sought to describe the socio-demographic features, pattern of COVID-19 spread and the viral load dynamics among subjects residing in northern, middle and part of the southern belt of Ghana.

**Methods:**

This was a cross-sectional retrospective study that reviewed records of samples collected from February to July, 2020. Respiratory specimens such as sputum, deep-cough saliva and nasopharyngeal swabs were collected from suspected COVID-19 subjects in 12 regions of Ghana for laboratory analysis and confirmation by real-time reverse transcription polymerase chain reaction (RT-PCR).

**Results:**

A total of 72,434 samples were collected during the review period, with majority of the sampled individuals being females (37,464; 51.9%). The prevalence of SARS-CoV-2 identified in the study population was 13.2% [95%CI: 12.9, 13.4). Males were mostly infected (4,897; 51.5%) compared to females. Individuals between the ages 21–30 years recorded the highest number of infections (3,144, 33.4%). Symptomatic subjects had higher viral loads (1479.7 copies/μl; IQR = 40.6–178919) than asymptomatic subjects (49.9; IQR = 5.5–3641.6). There was significant association between gender or age and infection with SARS-CoV-2 (p<0.05). Among all the suspected clinical presentations, anosmia was the strongest predictor of SARS-CoV-2 infection (Adj. OR (95%CI): 24.39 (20.18, 29.49). We observed an average reproductive number of 1.36 with a minimum of 1.28 and maximum of 1.43. The virus trajectory shows a gradual reduction of the virus reproductive number.

**Conclusion:**

This study has described the epidemiological profile of COVID-19 cases in northern, middle and part of the southern belt of Ghana, with males and younger individuals at greater risk of contracting the disease. Health professionals should be conscious of individuals presenting with anosmia since this was seen as the strongest predictor of virus infection.

## Introduction

Severe acute respiratory syndrome coronavirus-2 (SARS-COV-2), is the etiological agent for the COVID-19 disease which is currently a pandemic. This virus continues to spread globally with an average infection rate of 2.5, leaving over 20 million people infected with more than 700,000 associated deaths reported [1]. Although the infection could be asymptomatic, symptomatic signs may be mild, moderate severe or critical [2, 3]. Earlier reports showed a 26.4% risk of dying among individuals above 60 years and 4.1% of all deaths occurring in those below the age of 45 years [4]. Cases of COVID-19 in Africa has not been so alarming compared to the rest of the world. As of November 04, 2020, there were 1,814,642 reported cases in Africa which has resulted in over 40,000 deaths [5] compared to 9,383,979 and 10,866,134 cases resulting in 232,635 and 277,125 deaths in the United States (US) and Europe, respectively [5, 6]. Ghana confirmed her first two cases of COVID-19 on 12^th^ March, 2020 [7] and the case counts have risen to 48,643 with 320 deaths as of 4^th^ November, 2020 [8].

Various interventions including closure of borders, schools, mandatory wearing of masks and targeted lockdown have been introduced by the Government of Ghana in order to limit surges and the spread of infections. Although situational reports are usually provided by the Ministry of Health, detailed epidemiological data on cases are limited. To date, we do not have information on age and gender specific association with infection and the growth rate of the virus. The dynamism of viral load in different age groups, symptomatic and asymptomatic populations is also not well described. Measurement of viral load is indicative of active viral infection and replication. Higher viral load is associated with the onset of disease and it is a parameter for monitoring disease progression, treatment response, and relapse [9]. Zou et al., (2020) reported that among COVID-19 patients, both symptomatic and asymptomatic patients have similar viral load measurements [10]. The dynamics of the viral load distribution of the disease is however not fully understood in African populations. Correlations have been drawn between Middle East Respiratory Syndrome Coronavirus (MERS-CoV) disease distribution and gender with reports demonstrating higher disease incidence and severity among males [11]. This pattern is however yet to be explored in African populations.

The World Health Organization (WHO) has advised that decisions about this virus should be informed by evidence of data and science. It is therefore important that detailed epidemiological profile of those infected by the virus be provided in order to guide various interventions that could be implemented on the African continent. This study therefore aimed to describe the socio-demographic features, pattern or rates of virus infection through the period of this pandemic and viral load dynamism in subpopulations of SARS-CoV-2 infected individuals.

## Materials and methods

### Ethics statement

Ethical approval for this study was obtained from the Committee on Human Research, Publication and Ethics (CHRPE) of the School of Medicine and Dentistry, Kwame Nkrumah University of Science and Technology (KNUST) (CHPRE/AP/462/19) and the Ethical Review Committee of the Ghana Health Service (GHS-ERC087/03/20). Due to the retrospective nature of the study, informed consent was not obtained, and data were analyzed anonymously.

### Study area

**Fig 1:**
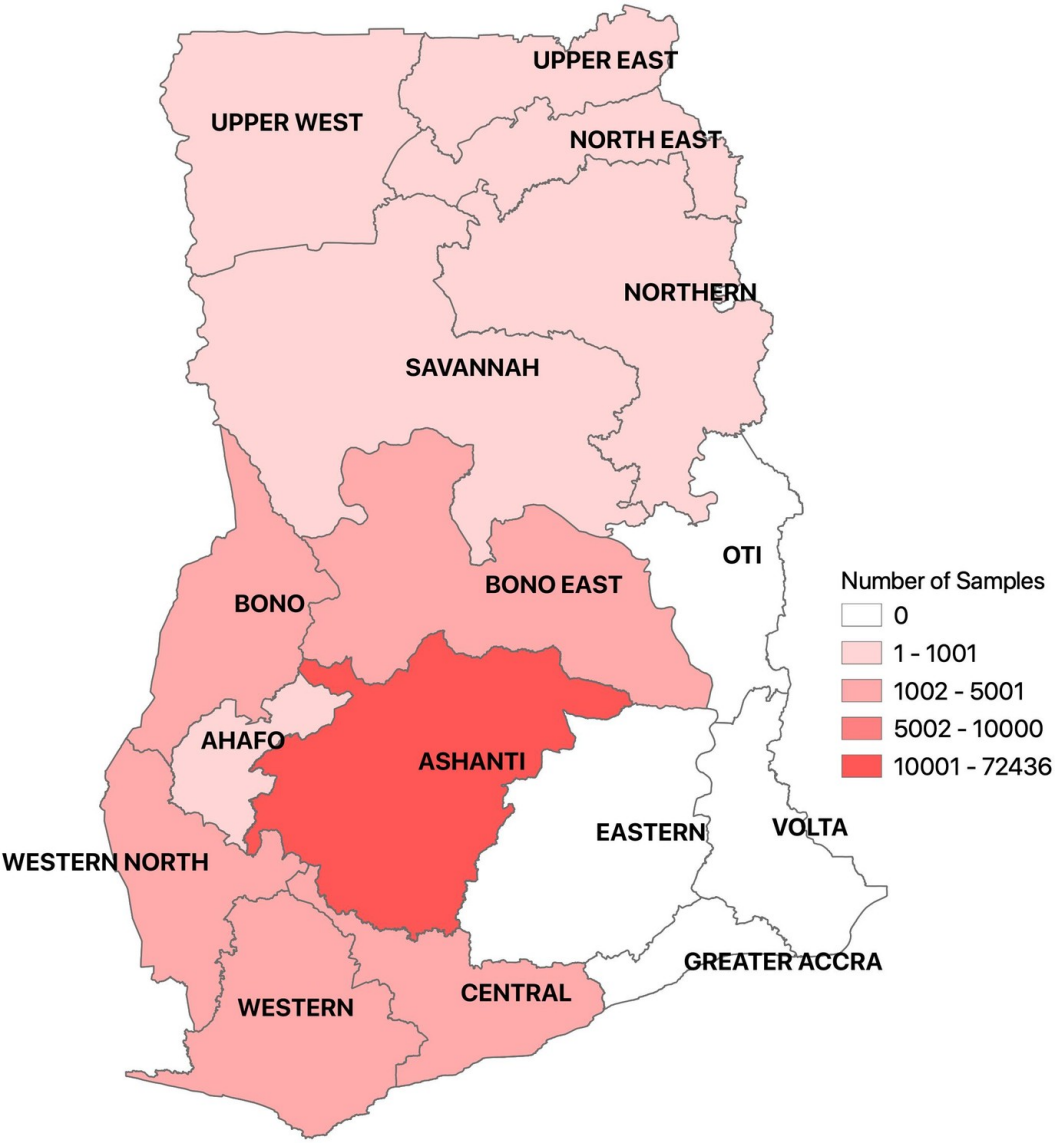
Description of sample sites.

This study was conducted at the Kumasi Centre for Collaborative Research in Tropical Medicine (KCCR) located on the campus of the Kwame Nkrumah University of Science and Technology (KNUST), Kumasi, Ghana. KCCR was established as a joint venture between the stakeholders of Ministry of Health of the Republic of Ghana, KNUST, and the Bernhard Nocht Institute of Tropical Medicine (BNITM) in Germany. It is currently the second largest testing site which serves the northern part of Ghana. During the early phase of the pandemic, KCCR received samples from 12 out of 16 regions of Ghana and tested approximately 1,200 samples on a daily basis. At the onset of the pandemic, samples were received from the Ashanti, Bono, Ahafo, Northern, Savannah, North East, Upper West, Upper East, Central, Western North, Western and Central regions of Ghana. These regions constitute 12 out of 16 regions in Ghana and cover the northern, middle and part of the southern belt. Fig 1 shows the regions where samples were collected.

### Study design and sample collection

This was a retrospective study carried out on subjects presenting with suspected COVID-19 from February 2020 to July, 2020. Samples were collected through routine surveillance and enhanced surveillance. Routine surveillance samples were collected from suspected subjects who presented with signs and symptoms of COVID-19 at the various health facilities based on defined criteria and in line with Ghana Surveillance Guidelines for the COVID-19 Outbreak. Enhanced surveillance samples were collected from primary/close contacts of COVID-19 cases and high-risk groups such as those living in crowded areas and slums. Respiratory tract samples such as sputum were collected from severely ill subjects. Asymptomatic subjects mainly produced saliva after a deep cough. Data and samples analyzed at KCCR are presented.

### Laboratory methods

All medical and laboratory staff were equipped with personal protective equipment (PPE) during collection and processing of samples. Samples were either collected in RNAlater (Qiagen, Hilden, Germany) for swabs or sterile container for sputum or deep-coughed saliva. All samples received were processed in a biosafety level 3 facility located within KCCR. Viral RNA was extracted using spin column-based kits from Da An Gene (Guangdong, China). Extractions were done following the manufacturer’s recommended protocol. Real-time polymerase chain reaction (RT-PCR) test was performed on the samples using Da An Gene PCR kits. The kit is designed to target the ORF1ab and nucleocapsid regions of the virus. Prior to using the kits, standardization and validation for detection of SARS-CoV-2 were performed using positive controls. All tests were ran using CFX96 Bio-Rad real-time PCR platform with CFX software version 1.6 (Bio-Rad, Singapore). The PCR cycling conditions were 50°C for 15 minutes for reverse transcription, 95°C for 1 minute for denaturation and 45 cycles of 94°C for 15 seconds and 55°C for 45 seconds. The analytical sensitivity of the kits was 500 copies/ml. All samples with threshold cycle (Ct) of 40 and above were considered as negative. Viral load was extrapolated from standard curve generated from plotting known viral concentrations against Ct values of a single target. All PCR runs were validated by the inclusion of positive and negative controls.

### Data collection

All samples received at KCCR came along with case-based forms. Epidemiological information including age, gender, symptoms and region were extracted from the case-based forms and entered into excel spreadsheet.

### Ethical approval

Ethical approval for this study was obtained from the Committee on Human Research, Publication and Ethics (CHRPE) of the School of Medicine and Dentistry, Kwame Nkrumah University of Science and Technology (KNUST) (CHPRE/AP/462/19) and the Ethical Review Committee of the Ghana Health Service (GHS-ERC087/03/20).

### Statistical analysis

Descriptive statistics were computed for continuous and categorical variables. For continuous variables; parameters such as medians with interquartile ranges were calculated for skewed data distributions and means were computed for normally distributed data. Proportions (percentages) were computed for categorical variables. Statistical comparisons between subgroup of continuous variables were evaluated by *t-*test, analysis of variance, Mann-Whitney U tests, and Kruskal-Wallis tests where appropriate. For purposes of analysis, we categorized levels of symptoms into moderate or mild and severe cases. Moderate or mild cases were individuals presenting with non-specific symptoms such as fever, fatigue, cough (with or without sputum production), anorexia, malaise, muscle pain, sore throat, dyspnea, nasal congestion, or headache. Severe cases were individuals presenting with mild symptoms in addition to having shortness of breath or breathlessness. Statistical analyses were performed using the R software package and p<0.05 based on a two-sided hypothesis was considered significant. The Map was drawn using Quantum GIS software (version 3.8) [12].

## Results

### Characteristics of subjects

A total of 72,436 study samples from subjects were tested between the period of 2^nd^ February, 2020 to 8^th^ July, 2020. Majority of the samples were collected in the Ashanti region of Ghana (63,127; 87.1% and the least from the Savannah region (15; 0.02%). Of all subjects, 37,464 (51.9%) were females. Samples from Ghanaians constituted 72,215 (99.8%) with asymptomatic subjects forming the majority (60,618; 84.3%). Of the 72,436 subjects reviewed, 6,035 (8.3%) had cough, 5,044 (7%) had headache, 2,615 (3.6%) had sore throat, 2,287 (3.2%) had runny nose and 2,014 (2.8%) had difficulty in breathing. The highest number of samples were from the 21-30-year (30.7%) group by contrast to the 81-119-year group (409; 0.6%) that registered the least number (Table 1).

**Table 1:** Socio-demographic and clinical characteristics of subjects.

	NEGATIVE	POSITIVE	Total	*p-value*
Total	62885	9549	72434	
^†^Gender of subjects				< 0.001
Males	29825 (47.6)	4897 (51.5)	34722 (48.1)	
Females	32842 (52.4)	4619 (48.5)	37461 (51.9)	
^†^Country of origin of subjects				< 0.001
Non-Ghanaian	163 (0.3)	4 (0)	167 (0.2)	
Ghanaian	62677 (99.7)	9534 (100)	72211 (99.8)	
^†^Categories of age groups				< 0.001
≤10	1452 (2.4)	280 (3)	1732 (2.4)	
11–20	7235 (11.7)	960 (10.2)	8195 (11.5)	
21–30	18671 (30.2)	3144 (33.4)	21815 (30.7)	
31–40	16535 (26.8)	2737 (29.1)	19272 (27.1)	
41–50	8651 (14)	1083 (11.5)	9734 (13.7)	
51–60	5291 (8.6)	690 (7.3)	5981 (8.4)	
61–70	2537 (4.1)	364 (3.9)	2901 (4.1)	
71–80	1014 (1.6)	110 (1.2)	1124 (1.6)	
81–119	363 (0.6)	46 (0.5)	409 (0.6)	
Abdominal pains				< 0.001
No	62663 (99.6)	9452 (99)	72115 (99.6)	
Yes	222 (0.4)	97 (1)	319 (0.4)	
Anosmia				< 0.001
No	62745 (99.8)	8836 (92.5)	71581 (98.8)	
Yes	140 (0.2)	713 (7.5)	853 (1.2)	
Breathing Difficulty				< 0.001
No	61525 (97.8)	8895 (93.2)	70420 (97.2)	
Yes	1360 (2.2)	654 (6.8)	2014 (2.8)	
Chest pains				< 0.001
No	62350 (99.1)	9285 (97.2)	71635 (98.9)	
Yes	535 (0.9)	264 (2.8)	799 (1.1)	
Cough				< 0.001
No	58894 (93.7)	7505 (78.6)	66399 (91.7)	
Yes	3991 (6.3)	2044 (21.4)	6035 (8.3)	
Diarrhoea				< 0.001
No	62505 (99.4)	9402 (98.5)	71907 (99.3)	
Yes	380 (0.6)	147 (1.5)	527 (0.7)	
Fever				< 0.001
No	60755 (96.6)	8031 (84.1)	68786 (95)	
Yes	2130 (3.4)	1518 (15.9)	3648 (5)	
Headache				< 0.001
No	59754 (95)	7636 (80)	67390 (93)	
Yes	3131 (5)	1913 (20)	5044 (7)	
Runny Nose				< 0.001
No	61547 (97.9)	8600 (90.1)	70147 (96.8)	
Yes	1338 (2.1)	949 (9.9)	2287 (3.2)	
Sneezing				< 0.001
No	62766 (99.8)	9427 (98.7)	72193 (99.7)	
Yes	119 (0.2)	122 (1.3)	241 (0.3)	
Sore Throat				< 0.001
No	61226 (97.4)	8593 (90)	69819 (96.4)	
Yes	1659 (2.6)	956 (10)	2615 (3.6)	

^†^Sub-categories contained missing values; hence, their total might not reflect the overall total of the study subjects.

### Distribution of SARS-CoV-2

**Fig 2:**
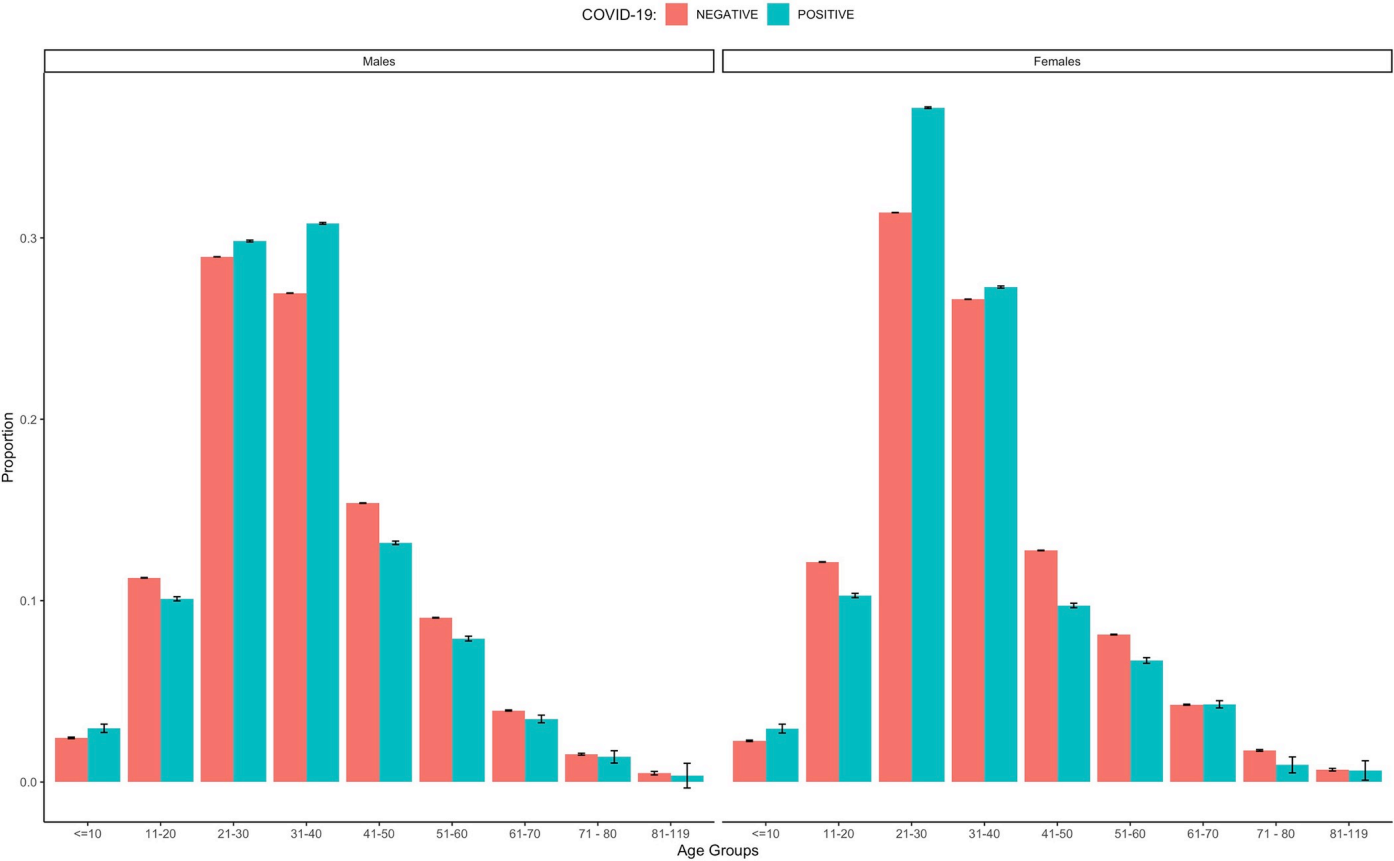
Age distribution of SARS-CoV-2 infections.

Table 1 gives details of virus distribution among the different types of variables collected. The prevalence of SARS-CoV-2 identified in the study populations was 13.2% [9549; 95%CI = 12.9% - 13.4.%). Males had higher number of infections (4,897; 51.5%) compared to females and the difference was statistically significant (p<0.001). Of all positive SARS-CoV-2 cases, the highest number of infections were recorded among age group 21–30 years (3,144, 33.4%) and the least occurred in age group 81–119 years (46; 0.5%). Most infections occurred in the age groups (21–40 years) as compared to older age groups (> 60 years) (Fig 2). There was significant association between gender or age group and infection with SARS-CoV-2 (p<0.05). Asymptomatic subjects had the highest number (5237; 56.8%) of infections compared to symptomatic persons. Among symptomatic individuals, abdominal pains, anosmia, breathing difficulty, chest pains, cough, diarrhoea, fever, headache, runny nose and sore throat were significantly associated with SARS-CoV-2 infection. We recorded a total of 24 deaths with three of this being positive for SARS-CoV-2. These samples were collected from patients who were already deceased.

**Fig 3:**
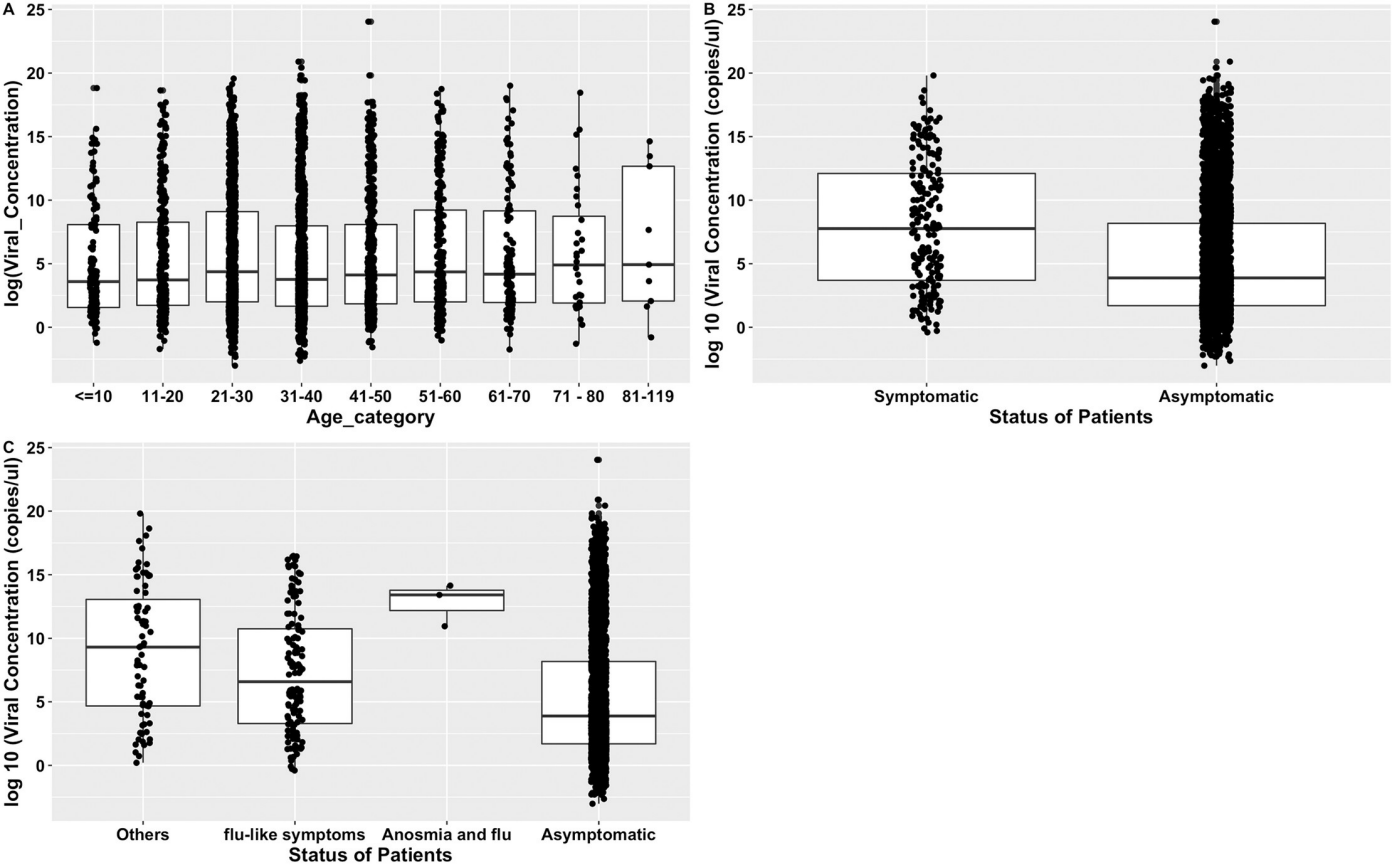
Viral load distribution in SARS-CoV-2 infected individuals.

Fig 3 describes variation in the viral loads for symptoms and age. A correlation analysis between the age and viral loads did not show any linear relationship (r = 0.01). However, there was significant variation in the viral loads for the different age categories. The least median viral load was found in subjects 10 years and below (36.4 copies/μl; IQR = 5.6–1502.9) and the highest was in those aged 71–80 years (409.1 copies/μl; IQR = 14.7–17,112). In terms of the symptom categories, symptomatic subjects had higher viral loads (1479.7 copies/μl; IQR = 40.6–178919) compared to asymptomatic subjects (49.9; IQR = 5.5–3641.6) and the difference was statistically significant (p<0.001). A breakdown of the symptomatic group showed significant differences in the viral loads with the highest occurring in individuals presenting with severe symptoms (1171 copies/μl (IQR = 35.4,105746.5), followed by those with moderate or mild symptoms (1131.7 copies/μl, IQR = 36.6,116824.5) and the least in asymptomatic subjects (42.6 copies/μl; IQR = 7.5–1988.7). The median difference was statistically significant (p<0.001).

### Risk factors associated with SARS-CoV-2 infection

Table 2 shows the independent risk factors of SARS-CoV-2 infection when a logistic regression model was fitted with infection status being the dependent variable. Individuals aged 10 years and below, 21–30 years and 31–40 years had higher odds of becoming infected with SARS-CoV-2 compared to the older age group (81–119 years). Similarly, being a male or non-Ghanaian increased one’s odds of infection with SARS-CoV-2. In terms of the clinical symptoms, subjects with anosmia, fever, headache, runny nose, sneezing and sore throat had higher odds of having SARS-CoV-2 infection. Anosmia was the highest predictor of COVID-19 disease (Adj. OR (95%CI): 24.39 (20.18, 29.49).

**Table 2:** Risk factors associated with SARS-CoV-2 infection.

Variables	Crude OR (95% CI)	Adj. OR (95%CI)	P (Walds test)
**Age Category Ref = 81–119 years**			
**≤10**	**1.31 (0.95,1.82)**	**1.75 (1.25,2.46)**	**0.001**
11–20 years	1.02 (0.74,1.39)	1.38 (0.99,1.91)	0.057
**21–30 years**	**1.27 (0.94,1.74)**	**1.71 (1.24,2.36)**	**0.01**
**31–40 years**	**1.26 (0.92,1.71)**	**1.69 (1.22,2.34)**	**0.001**
41–50 years	0.96 (0.7,1.31)	1.32 (0.95,1.83)	0.1
51–60 years	0.97 (0.71,1.34)	1.2 (0.82,1.75)	0.128
61–70 years	1.12 (0.81,1.55)	1.37 (0.97,1.92)	0.073
71–80 years	0.84 (0.58,1.21)	0.89 (0.61,1.3)	0.534
**Gender: Males vs Females**	**1.17 (1.12,1.22)**	**1.17 (1.12,1.23)**	**< 0.001**
**Country of Origin**	**5.96 (2.22,16.03)**	**9.15 (3.36,24.93)**	**< 0.001**
**Ghanaian vs Non-Ghanaian**			
**Clinical Symptoms**			
Abdominal Pains Yes vs No	2.93 (2.3,3.73)	0.85 (0.65,1.12)	0.256
**Anosmia: Yes vs No**	**35.85 (29.85,43.07)**	**24.39 (20.18,29.49)**	**< 0.001**
Diarrhoea: Yes vs No	2.58 (2.12,3.12)	0.85 (0.68,1.07)	0.161
**Fever: Yes vs No**	**5.46 (5.09,5.86)**	**2.59 (2.37,2.82)**	**< 0.001**
**Headache: Yes vs No**	**4.8 (4.51,5.11)**	**2.56 (2.37,2.77)**	**< 0.001**
**Runny Nose: Yes vs No**	**5.14 (4.71,5.61)**	**1.98 (1.79,2.2)**	**< 0.001**
**Sneezing: Yes vs No**	**6.77 (5.24,8.73)**	**2.62 (1.95,3.51)**	**< 0.001**
**Sore Throat: Yes vs No**	**4.11 (3.78,4.47)**	**1.42 (1.28,1.57)**	**< 0.001**

### Epidemiology profile of cases

**Fig 4:**
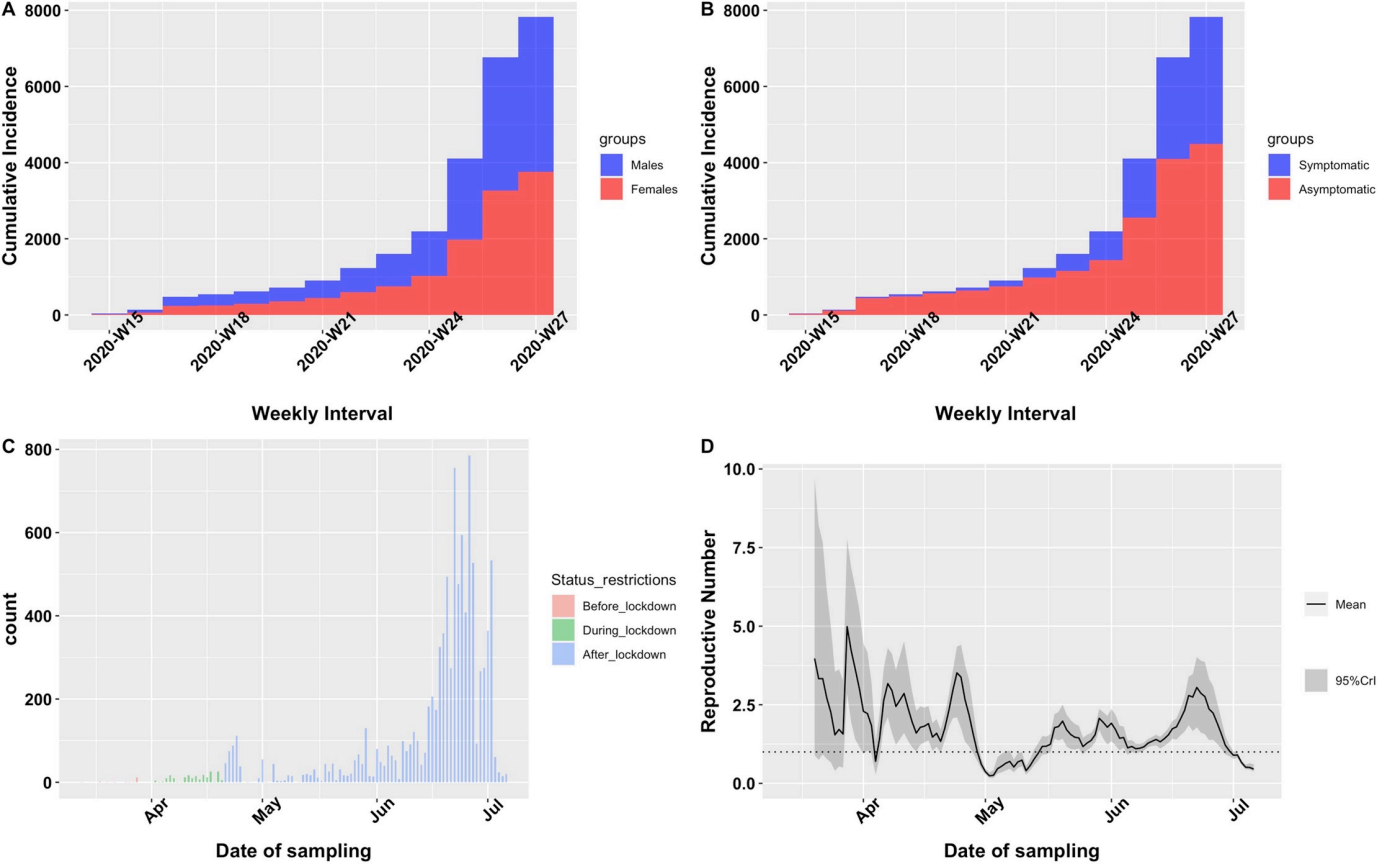
Epidemiological profile of SARS-CoV-2 infections.

Fig 4 provides information on the weekly cumulative incidence stratified by gender (males versus females) and symptom status (symptomatic versus asymptomatic). Following the first reported case of SARS-CoV-2 infection on the 12^th^ of March 2020, there was a weekly increase in number of new infections. Similar increases in infections were observed for both males and females (Fig 4A). Asymptomatic cases were much more predominant in the early period of the outbreak but symptomatic cases eventually became much more common as the pandemic progressed (Fig 4B).

The impact of a suppressive intervention on the rate of infections was also examined. One major intervention adopted by the country was imposition of lockdown which was announced on 30^th^ of March, 2020. This was followed by a period of some easing of restrictions 3 weeks afterwards. In order to compare the virus proportions before, during and after lockdown, we created a three-level categorical variable for the period before lockdown (29/2/2020-30/03/2020), during lockdown (31/03/2020–20/04/2020) and after lockdown (08/07/2020). The virus positivity rate was 6.1% (20/324) before lockdown, 0.85% (195/22716) during lockdown and 23.4% (9334/39845) after lockdown (Fig 4C). This difference in proportion was significant (p<0.001). In order to understand the growth rate of the virus, we estimated the daily reproductive number (R_0_) using log-linear models to parameterize a discrete gamma distribution for the serial interval. Estimates were made using a mean serial interval of 7.5 days and standard deviation of 3.4 days (Lit et., 2020). We found the mean R_0_ to be 1.36 with a minimum of 1.28 and maximum of 1.43. A plot of the instantaneous effective R_0_ showed multiple peaks possibly depicting surges in infections in particular hotspots. The highest peak of around 5 was observed during the lockdown period and the very recent R_0_ of around 3 occurred in June during the easing phase of restrictions (Fig 4D).

## Discussion

Human coronaviruses have contributed significantly to the burden of respiratory tract infections globally. Prior to the SARS-CoV and MERS-CoV outbreaks in 2003 and 2012, respectively, coronaviruses were thought to cause mild, self-limiting respiratory infections in humans [13, 14]. However, occurrence of these outbreaks and subsequent emergence of the current COVID-19 pandemic have changed the narrative. This study showed the cumulative detection of 9,549 (13.2% of tested specimens) SARS-CoV-2 in the study areas examined. The observed positivity rate was higher than the current national prevalence of 9.7% and reflects active transmission phase of the virus in these regions. Sustained person to person transmission was ongoing with localized outbreaks and possible surges in hotspots.

Although, majority of the sampled individuals were females (51.9%), a higher proportion of those who tested positive were males. This is comparable to other studies with high proportions of SARS-CoV-2 infection in males [15–19]. This male preponderance to infection has been explained by some studies that mast cells in females are able to trigger a more active immune response, which may help them fight infectious diseases better than males [18]. Furthermore, other genetic components such as the X chromosomes and hormones, typically estrogens, both predominantly found in females have been linked to provide some significant level of protection against SARS-CoVs [18, 20]. Interestingly, this study showed that older individuals (>60 years old) were less likely to be infected with COVID-19 than the younger groups (Table 2). One reason for this observation could be that the young groups are actively working and outdoor engagements were rampant, with little adherence to safety protocols, resulting in increased cases. This observation agrees with data from Brazil [21] and suggests that younger persons could be major drivers of SARS-CoV-2 infections. Notwithstanding, all age groups are at risk of contracting COVID-19, and in terms of disease severity, older individuals are at a greater risk due to physiological changes that come with ageing, compromised immune system and potential underlying health conditions [22].

Our study also measured the viral loads of subjects whose samples were analyzed at KCCR in Kumasi. The viral load profile is important for guiding antiviral treatment and could explain the pattern of recovery and transmission of infections. Unlike other studies which showed children had higher loads of the virus than adults [23], our study identified a higher load of the virus in elderly persons compared to younger persons. The finding is consistent with previous reports of SARS-CoV which suggest older age was an independent factor associated with higher viral load and that these could contribute to impairment of the innate and adaptive immune responses and increase the risk of death [24]. This phenomenon could explain why older persons are dying more in Africa than younger age groups.

An evaluation of the viral loads between symptomatic and asymptomatic subjects was also analyzed. Symptomatic subjects had higher loads of the virus than asymptomatic subjects. For symptomatic subjects, those with severe symptoms had higher loads than those with mild symptoms. There are mixed reports of variation in viral loads of symptomatic and asymptomatic populations. Lavezzo *et al*., reported that there was no difference in viral loads for symptomatic and asymptomatic subjects compared in the Italian city of Vo [25]. Our findings corroborate a report by Liu *et al*., which observed higher mean viral load in subjects with severe symptoms compared to mild clinical presentations [26].

In order to slow down the rapid spread of the virus, several interventions were adopted and implemented by the Government of Ghana. Of note was the imposition of partial lockdown in some major cities in the country for 3 consecutive weeks. We observed the rate of positivity of the virus was very low (0.85%) during the lockdown period compared to the period before (6.1%) and after (23.4%) the lockdown intervention. Although the difference in virus detection was significant, it was unclear whether this was as a result of the lock-down intervention. The low prevalence observed during the lockdown period could be as a result of an enhanced contact tracing of large populations most of whom were asymptomatic. Most of the testing performed before and after the lock-down period targeted symptomatic individuals and thus more likely to yield positive results. Some authors have reported imposition of lockdowns as leading to significant reduction in infections [27] but this evaluation would require an adequate and consistent sampling and testing approach.

In COVID-19 patients, the main clinical manifestation is fever and cough often characterized by lymphocytopenia and ground-glass opacity changes on chest computed tomography [28]. However, due to the evolving nature of COVID-19, information regarding symptoms is also evolving. Apart from fever, cough, sore throat and other flu-like symptoms, this study identified anosmia (loss of smell) as the strongest predictor of SARS-CoV-2 infection, and this evidence is consistent with other studies reported elsewhere [29–31]. One possible reason could be because of the expression of SARS-CoV-2 receptors (ACE_2_ and TMPRSS2) in the nasal respiratory epithelium and olfactory sensory epithelium which enables the virus to cause injury to the cells. More recently, an association between COVID-19 and altered olfactory function has been reported in South Korea, Iran, Italy, France, UK and the United States [32–34].

We identified 3 SARS-CoV-2 related deaths during the study period. This could be underestimated and may not reflect the number of deaths in the study areas due to limited data availability. We noted that most of the samples were collected from patients who had passed on and for which the hospital management wanted to rule out COVID-19 associated deaths. Deaths which occurred after laboratory samples tested positive were not reported back to our Centre. A large number of deaths from COVID-19 pandemic have occurred in the United States, China, the United Kingdom, Italy, Iran and Spain, with relatively fewer deaths recorded in Africa [35]. The relatively lower death toll of COVID-19 in Africa may be due to differences in genetic or climatic factors, or perhaps the impact is yet to be felt [35]. Studies have shown that comorbidities have the tendency of increasing individual’s susceptibility to COVID-19, especially among the elderly [36]. Chronic non-communicable diseases have been noted to have significant influence on COVID-19 prognosis, although these conditions account for a relatively lighter burden in Africa compared to developed countries [35]. Many of the poorer outcomes, including increased mortality due to COVID-19 have been related to cardiovascular comorbid conditions [37–39]. However, a large multicentre study conducted in Italy contrasts this finding and reports on factors such as age, renal impairment and high C-reactive protein levels to have strong predictive roles for mortality [40]. Another important aspect to look out for especially in sub-Sahara Africa is the occurrence of COVID-19 and malaria incidence. Gennaro *et al*., reported that malaria and COVID-19 present similar aspects and this seems to have a strong potential for mutual influence [41]. Healthcare professionals through the ministries of health and national malaria control programs are advised to ensure that malaria control efforts are not disrupted while dealing with the COVID-19 response [42, 43].

One limitation of this study was our inability to test other known respiratory viruses among the subjects. This is particularly important because negative results for SARS-CoV-2 does not necessarily rule out the possibility of other respiratory pathogens. Future studies could explore the role of other clinically relevant viruses among subjects with respiratory tract infections.

## Conclusion

We have demonstrated high cumulated prevalence of COVID-19 in northern, middle and part of the southern belt of Ghana is high, with males and younger individuals at greater risks of contracting the disease. Clinicians should be mindful of the symptom of anosmia especially in outpatients in order to minimize delays in the diagnosis of COVID-19.

## Supporting information

S1 File (XLSX)

S2 File (XLSX)
